# Putative correlates of protection against shigellosis assessing immunomarkers across responses to *S. sonnei* investigational vaccine

**DOI:** 10.1038/s41541-024-00822-2

**Published:** 2024-03-08

**Authors:** Valentino Conti, Omar Rossi, Kristen A. Clarkson, Francesca Mancini, Usman N. Nakakana, Eleanna Sarakinou, Andrea Callegaro, Pietro Ferruzzi, Alessandra Acquaviva, Ashwani Kumar Arora, Elisa Marchetti, Francesca Necchi, Robert W. Frenck, Laura B. Martin, Robert W. Kaminski, Audino Podda, Francesca Micoli

**Affiliations:** 1 GSK Vaccines Institute for Global Health, Siena, Italy; 2 Department of Diarrheal Disease Research, Bacterial Diseases Branch, Walter Reed Army Institute of Research, Silver Spring, MD USA; 3 GSK, Rixensart, Belgium; 4 Division of Infectious Diseases, Cincinnati Children’s Hospital Medical Center, Cincinnati, OH USA; 5 Present Address: Horizon Therapeutics, Deerfield, IL USA; 6 Present Address: Independent Consultant, Siena, Italy; 7 Present Address: US Pharmacopeial Convention, Rockville, MD USA; 8 Present Address: Latham BioPharm Group, Cambridge, MA USA

**Keywords:** Vaccines, Drug development

## Abstract

*Shigella* spp. are a leading bacterial cause of diarrhea. No widely licensed vaccines are available and there is no generally accepted correlate of protection. We tested a *S. sonnei* Generalized Modules for Membrane Antigen (GMMA)-based vaccine (1790GAHB) in a phase 2b, placebo-controlled, randomized, controlled human infection model study (NCT03527173) enrolling healthy United States adults aged 18–50 years. We report analyses evaluating immune responses to vaccination, with the aim to identify correlates of risk for shigellosis among assessed immunomarkers. We found that 1790GAHB elicited *S. sonnei* lipopolysaccharide specific α4β7+ immunoglobulin (Ig) G and IgA secreting B cells which are likely homing to the gut, indicating the ability to induce a mucosal in addition to a systemic response, despite parenteral delivery. We were unable to establish or confirm threshold levels that predict vaccine efficacy facilitating the evaluation of vaccine candidates. However, serum anti-lipopolysaccharide IgG and bactericidal activity were identified as potential correlates of risk for shigellosis.

## Introduction

*Shigella* is a major cause of diarrheal disease worldwide, with a broad geographical distribution and affecting all age groups. Mortality due to *Shigella* remains substantial in children under five years of age in low- and middle-income countries (LMICs)^1^. Additionally, shigellosis occurring early in childhood is associated with persistent diarrhea, post-infectious sequelae, and linear growth faltering^2,3^.

There are four *Shigella* species, two of which are dominant: *S. flexneri* (with 15 serotypes, 2a being the most prevalent) and *S. sonnei*. *S flexneri* is the most common species in LMICs whereas *S. sonnei* is the leading species in high-income countries^1,4,5^. Increasing antibiotic resistance for *Shigella* is a growing concern^4,6,7^, emphasizing the need for a vaccine to prevent shigellosis. Despite many attempts, no effective vaccine candidate against *Shigella* is widely available^8^. Several investigational vaccines based on various technologies are under development, mainly targeting the O Antigen (OAg) moiety of the lipopolysaccharide (LPS)^9^ which is known to play a key role in naturally-acquired and vaccine-elicited immunity against shigellosis^10^.

Vaccine development and clinical evaluation are complicated by the lack of an established correlate of protection (CoP) against *Shigella*. It is widely accepted that the development of anti-LPS serum immunoglobulin (Ig) G antibodies can confer protection against shigellosis^10–12^. More recently, the role of anti-LPS serum IgG as a CoP has been reinforced: in a re-analysis of serologic and vaccine efficacy (VE) data from a trial of the *S. sonnei* OAg-*Pseudomonas aeruginosa* recombinant exoprotein A glycoconjugate vaccine, a threshold of ≥1:1600 titer was associated with reduced risk of shigellosis and predicted VE against *S. sonnei* shigellosis in young Israeli adults^13^. Other potential CoPs include functional serum antibodies, mucosal antibodies produced by gut-homing lymphocytes, antibody-secreting cells (ASCs), and antibodies secreted in culture supernatants by lymphocytes isolated from peripheral blood mononuclear cells (PBMC) collected 7–10 days following exposure to *Shigella*^14–21^.

**Fig. 1:**
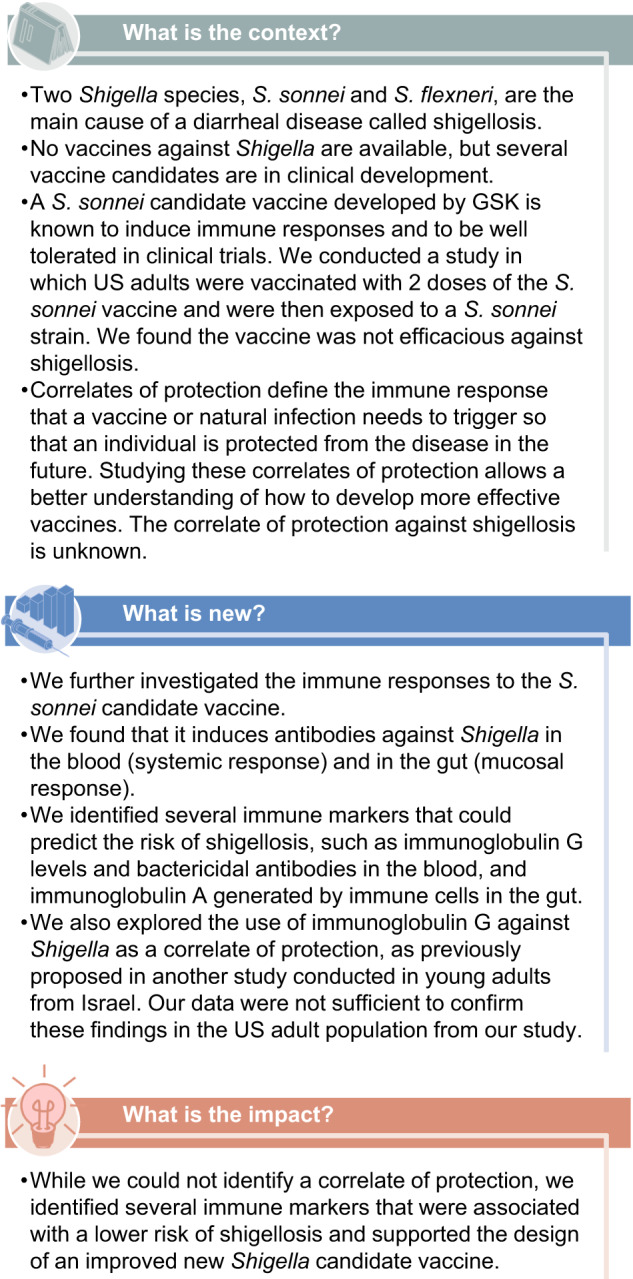
The plain language summary (PLS). Figure showing the research context, what is new, and the impact of the research.

The Generalized Modules for Membrane Antigens (GMMA) are outer membrane particles derived from genetically modified Gram-negative bacteria that have been proposed as a platform for OAg delivery^22–24^. *Shigella* GMMA are inherently released by bacteria mutated to increase yields and present attenuated endotoxicity through the modification of the lipid A moiety of LPS^25,26^. We have developed an investigational *S. sonnei* vaccine (1790GAHB) based on this technology^27^. 1790GAHB was shown to have an acceptable safety profile^28^ and induce bactericidal anti-LPS IgG responses in adults from both endemic^29,30^ and non-endemic settings^18,31,32^. We previously reported the results of a phase 2b, randomized, controlled human infection model (CHIM) study conducted in adults from the United States (US), in which participants aged 18–50 years received two doses of either 1790GAHB or placebo, 28 days apart and were challenged with *S. sonnei* strain 53G at day (D) 57 after the first vaccination^33^. 1790GAHB did not demonstrate clinical efficacy against shigellosis. However, baseline and pre-challenge anti-*S. sonnei* LPS serum IgG antibody levels and serum bactericidal activity (SBA) were higher in participants who did not develop shigellosis post-challenge, indicating their role in clinical protection^33^. Here, we further describe the immune responses elicited by 1790GAHB and evaluate the association of various immunological markers with protection from shigellosis, with the aim to identify correlates of risk (CoRs; defined as immunomarkers for which immune responses are associated with protection against clinical outcome)^34,35^ for shigellosis. We also explored the role of anti-*S. sonnei* LPS serum IgG as a CoP in the CHIM trial population of US adults. A summary contextualizing the results and potential clinical relevance and impact of the research is provided in the plain language summary (Fig. 1).

## Results

### Demographics

**Fig. 2:**
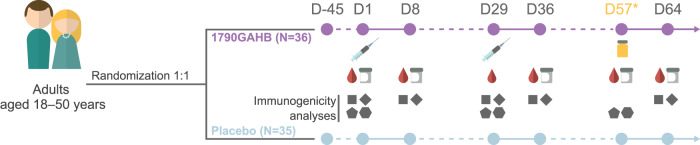
Study scheme. Study design showing the study population, randomization, and vaccination scheme. All subjects in per protocol analysis received two vaccinations at Day 1 and Day 29, received the challenge agent on day 57, with relevant blood draws for immunogenicity pre and post vaccination. D day, N number of participants. The syringe represents injection with 1790GAHB or placebo. The blood drop and gray container represent blood and stool samples, respectively.

A total of 71 adults were enrolled and vaccinated in the CHIM trial with 36 participants receiving 1790GAHB and 35 receiving placebo (Fig. 2).

At D57 (28 days after the second vaccination), 33 and 29 participants in the 1790GAHB group and the placebo group, respectively, received a challenge dose consisting of ~1500 colony-forming units of reconstituted lyophilized *S. sonnei* strain 53G, developed by the Walter Reed Army Institute of Research (Silver Spring, Maryland, US)^36^. Reasons for elimination from the per-protocol sets were previously reported^33^; demographic characteristics are summarized in Supplementary Table 1.

### Immune responses in the 1790GAHB versus the placebo group

Anti*-S. sonnei* LPS-specific serum IgG levels and SBA responses were previously reported in detail. Briefly, in the 1790GAHB group, anti-*S. sonnei-specific* LPS serum IgG geometric mean concentrations increased 2.33- and 5.19-fold at D8 (7 days after first vaccination) and D57 (28 days after second vaccination), respectively, compared with baseline; no increase was observed in the placebo group. At D57, SBA geometric mean titers increased 2.09-fold in the 1790GAHB group and 1.41-fold in the placebo group compared to baseline^33^.

**Fig. 3:**
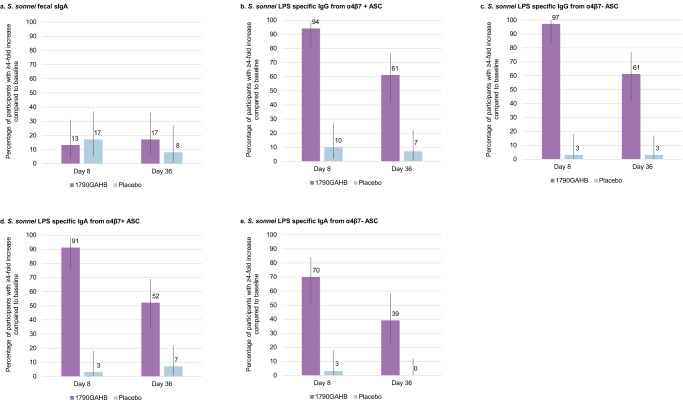
Percentage of participants with ≥4-fold increase in levels of *S. sonnei* LPS fecal sIgA and *S. sonnei* LPS specific IgG/IgA levels from α4β7+ and α4β7− ASCs. Bar graph showing from (**a**) to (**e**), *S.sonnei* fecal sIgA (**a**); *S.sonnei* LPS specific IgG from α4β7+ ASCs (**b**); *S.sonnei* LPS specific IgG from α4β7− ASCs (**c**); *S.sonnei* LPS specific IgA from α4β7+ ASCs (**d**); and *S.sonnei* LPS specific IgA from α4β7− ASCs (**e**). The 1790GAHB group shows a higher percentage of participants (represented by higher bars) achieving a 4-fold increase or more from baseline for all the parameters, at both Day 8 and Day 36, except for fecal IgA which was similar between recipients of 1790GAHB and placebo at both timepoints. The 95% confidence intervals are represented by the vertical overlay error bars in each case.

Four (13%) and 5 (17%) participants in the vaccine group and 4 (17%) and 2 (8%) participants in the placebo group showed a ≥4-fold increase in *S. sonnei* LPS fecal secretory immunoglobulin A (sIgA) titers at D8 and D36 (7 days after second vaccination), respectively (Fig. 3a). However median values did not substantially increase at post-vaccination timepoints for either the 1790GAHB or the placebo group (Supplementary Table 2).

The percentage of participants with ≥4-fold increase in IgG/IgA levels from α4β7+/α4β7− ASCs was higher in the 1790GAHB group compared to placebo recipients. Thirty-one (94%) and 20 (61%) participants in the 1790GAHB group versus 3 (10%) and 2 (7%) participants in the placebo group had ≥4-fold increase in IgG levels from α4β7+ ASCs at D8 and D36, respectively (Fig. 3b). Thirty-two (97%) and 20 (61%) participants in the 1790GAHB group versus 1 (3%) and 1 (3%) participant in the placebo group had ≥4-fold increase in IgG levels from α4β7− ASCs at D8 and D36, respectively (Fig. 3c). Thirty (91%) and 17 (52%) 1790GAHB recipients versus 1 (3%) and 2 (7%) placebo recipients for IgA levels from α4β7+ ASCs and 23 (70%) and 13 (39%) 1790GAHB recipients versus 1 (3%) and 0 (0%) placebo recipients for IgA levels from α4β7− ASCs had ≥4-fold increases at D8 and D36, respectively (Fig. 3d, e)

Higher median *S. sonnei* LPS specific IgG/IgA levels from α4β7+ and α4β7− ASC were observed in the 1790GAHB group than in the placebo group at D8 and D36 (Supplementary Table 2). When stratified by outcome, the percentage of participants with ≥4-fold increases in *S. sonnei* LPS specific IgG/IgA levels from α4β7+ and α4β7− ASC were overall similar between participants developing or not developing shigellosis post-challenge, as shown by overlapping 95% confidence intervals (CIs) (Supplementary Fig. 1).

SBA against OAg-positive and OAg-negative *S. sonnei* strains was assessed after LPS-specific antibody depletion. For the OAg-positive *S. sonnei* strain, no residual bactericidal activity was detected in sera depleted of anti-LPS IgG antibodies compared to non-depleted sera of 1790GAHB vaccinees (previously reported^33^; Table 1). Depleted sera were bactericidal against the OAg-negative strain and SBA titers tended to be higher in the vaccine than in placebo recipients (Supplementary Table 2).

**Table 1:** Serum bactericidal activity against OAg-positive *S. sonnei* strain for sera depleted and non-depleted of LPS specific antibodies, in participants vaccinated with 1790GAHB (per protocol immunogenicity set)

	Depleted sera		Non-depleted sera^33^	
	*N*	GMT (95% CI)	*N*	GMT (95% CI)
Day 1	34	16.50 (16.50–16.50)	32	81.95 (57.27–117.26)
Day 29	34	16.50 (16.50–16.50)	32	204.65 (113.56–368.80)
Day 57	34	16.50 (16.50–16.50)	32	171.11 (99.57–294.04)

The lower limit of quantification (LLOQ) for the assay used to assess S. sonnei bactericidal titers after LPS-specific antibodies depletion was an inhibition concentration 50 (IC50) of 33. Titers below the LLOQ were set to half that limit for the purpose of the analysis.

*OAg* O antigen, *LPS* lipopolysaccharide, *N* number of participants, *GMT* geometric mean titer, *CI* confidence interval.

**Fig. 4:**
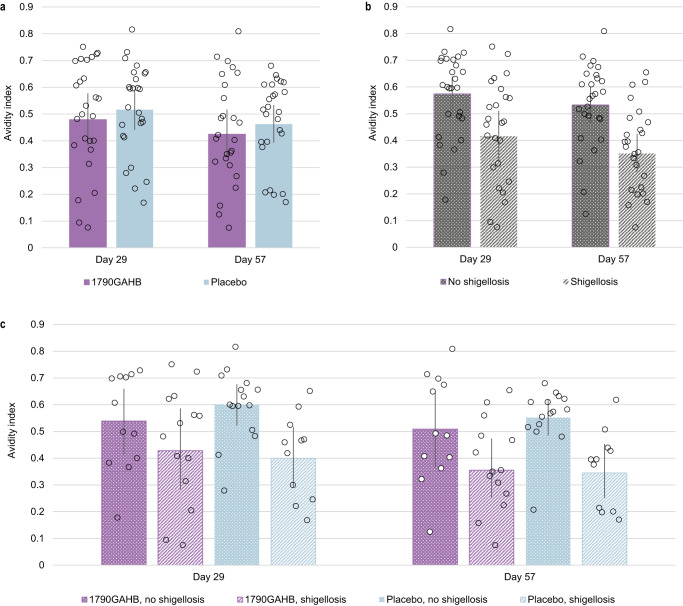
*S. sonnei* LPS specific total IgG avidity at 28 days post-first and second vaccination, in samples grouped by treatment, outcome and both, post-challenge dose. Bar chart showing the avidity index on the y axes, at Day 29 and Day 57, of participants grouped by **a** treatment i.e. who received 1790GAHB or placebo; **b** outcome, i.e. who did not develop, and developed shigellosis, and **c** combination of both. The bars representing participants who did not develop shigellosis were higher than those for participants with shigellosis, both among 1790GAHB and placebo recipients. Gray points represent individual participant values’ and the 95% confidence intervals are represented by the vertical overlay line in each case. LPS, lipopolysaccharide; IgG immunoglobulin G.

The avidity of anti-*S. sonnei* LPS antibodies was also evaluated. The avidity index, expressed as the ratio of IgG concentration determined by enzyme-linked immunosorbent assay (ELISA) in the presence or absence of chaotropic agent for the same sample, was similar for participants who received 1790GAHB or placebo, at D29 and D57 (Fig. 4a). Mean antibody avidity at D29 and D57 tended to be higher in participants who did not develop shigellosis post-challenge (Fig. 4b). When participants were grouped both by treatment and challenge outcome, the mean avidity index of participants who did not develop shigellosis was higher (with non-overlapping CIs of the means) compared to that of participants with shigellosis in the placebo group at both D29 and D57; in contrast, only a trend was observed in the 1790GAHB group (Fig. 4c).

**Fig. 5:**
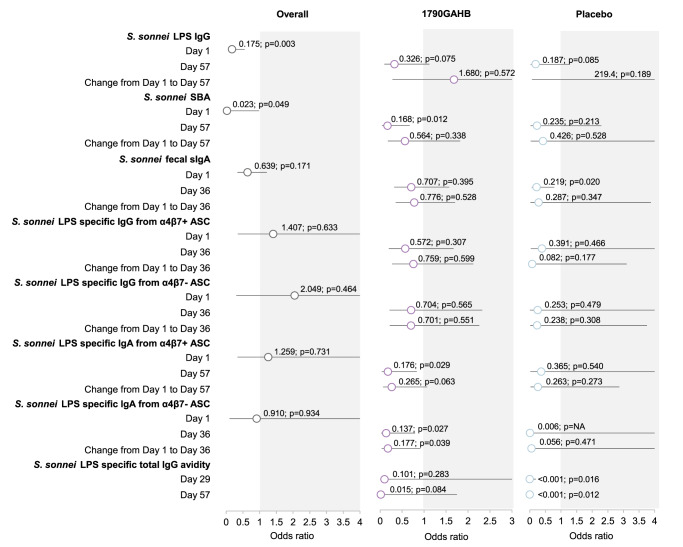
Correlation of risk analysis: association between assessed immunomarkers and risk of shigellosis, by timepoint and group. Graphical representation of correlate of risks: for each immunomarker, association with risk of developing shigellosis is assessed, by timepoint and by change between timepoints in individual rows; overall and among recipients of 1790GAHB and placebo in the columns. Odds ratios of shigellosis are represented (circles) along with 95% confidence intervals (horizontal bars). Odds ratio values < 1 (white area in the graphs) indicate that higher values of immunomarker are associated with lower risk of shigellosis The *p*-values show if association between immunomarker and shigellosis is statistically significant (i.e. horizontal bar not crossing the gray area).

Correlates of risk are identified using the results of logistic regression models as summarized in Fig. 5 and Supplementary Table 3; odds ratio (OR) < 1 indicate that higher values were associated with lower risk of shigellosis. As no VE was observed^33^, data for the two groups were pooled at D1 (pre-vaccination), to ensure a higher power to detect association between each immunomarker and shigellosis outcome.

Higher *S. sonnei* LPS-specific IgG levels (OR = 0.175; *p* = 0.003) at D1 were strongly associated with lower risk of shigellosis, while SBA levels (OR = 0.023; *p* = 0.049) showed borderline association. None of the other immunomarkers showed association with a lower risk of shigellosis at baseline (Fig. 5).

Increases in *S. sonnei* LPS-specific IgG levels and SBA from D1 to D57 and higher *S. sonnei* LPS-specific IgG levels at D57 were not associated with a reduced risk of shigellosis. The OR for shigellosis was <1 for SBA levels against *S. sonnei* at D57 in the 1790GAHB group (OR = 0.168, *p* = 0.012).

For higher *S. sonnei* LPS-specific fecal sIgA levels, the OR was <1 in the placebo group at D36 (OR = 0.219, *p* = 0.020), but not in the 1790GAHB group (Fig. 5).

In participants vaccinated with 1790GAHB, ORs < 1 were observed for higher *S. sonnei* LPS-specific IgA levels from α4β7+/α4β7− ASCs at D36 (OR = 0.176, *p* = 0.029/ OR = 0.137, *p* = 0.027), as well as their increase from D1 to D36 (OR = 0.265; *p* = 0.063/ OR = 0.177; *p* = 0.039). No association was observed for *S. sonnei* LPS-specific IgG levels from α4β7+/α4β7− ASCs (Fig. 5).

There was a clear association between higher *S. sonnei* LPS specific IgG avidity and protection in the placebo group at D29 (OR < 0.001; *p* = 0.0162) and D57 (OR < 0.001; *p* = 0.0121), but not in the 1790GAHB group (Fig. 5).

### Anti-S. sonnei LPS specific serum IgG levels as correlate of protection in US adults

Immunomarker-derived VE (VE_IM_) was calculated using data from the US adults in the CHIM study, based on the proportion of participants vaccinated with 1790GAHB with *S. sonnei* LPS serum IgG levels below the protection threshold of 1:1600 proposed by Cohen et al.^13^. A cut-off of 396 ELISA units (EU)/mL in the GSK Vaccines Institute for Global Health (GVGH) assay was established as the equivalent of this threshold from the Tel Aviv University (TAU) assay. This cut-off did not completely predict protection as 5/27 (18.5%) of participants from the pooled 1790GAHB and placebo groups who developed shigellosis had anti-*S. sonnei* LPS serum IgG ≥396 EU/mL at the pre-challenge timepoint (D57). The IgG threshold showed a sensitivity (probability of correctly identifying infected participants) of 81.5% and a specificity (probability of correctly identifying protected participants) of 33.3% (Table 2).

**Table 2:** Distribution of shigellosis cases and non-cases from the CHIM study population according to *S. sonnei* LPS serum IgG threshold at 28 days post-second vaccination

	Shigellosis outcome			
Threshold	Cases	Non-cases	Sensitivity	Specificity
<396 EU/mL	22	22	81.5%	
≥396 EU/mL	5	11		33.3%
Total	27	33		

396 EU/mL in the current GSK Vaccines Institute for Global Health enzyme-linked immunosorbent assay used is equivalent to the 1:1600 titer in the Tel Aviv University assay^13^.

*CHIM* controlled human infection model, *LPS* lipopolysaccharide, *IgG* immunoglobulin G, *EU* enzyme-linked immunosorbent assay unit.

Sensitivity was calculated as the number of participants with shigellosis and *S. sonnei* LPS serum IgG levels below the threshold over the total number of participants with shigellosis. Specificity was defined as the number of participants without shigellosis and *S. sonnei* LPS serum IgG levels equal to or above the threshold over the total number of participants without shigellosis.

**Fig. 6:**
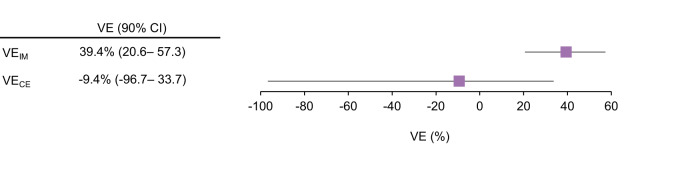
Forest plot of immunomarker-derived vaccine efficacy (VE_IM_) and vaccine efficacy against clinical event (shigellosis) (VE_CE_) estimated in the CHIM study population. Graphical representation of Immunomarker-derived VE (VE_IM_) calculated using the IgG protection threshold proposed by Cohen et al.^13^. and vaccine efficacy against observed clinical event (VE_CE_), represented by squares with their corresponding 90% confidence intervals (horizontal lines).

Over the 8-day post-challenge period, VE_IM_ (i.e., VE calculated using the IgG protection threshold) was 39.4% (90% CI 20.6–57.3). VE_IM_ and the VE observed for the primary clinical event (VE_CE_) previously reported^33^ were not comparable (39.4% versus −9.4%), although 90% CIs overlapped (Fig. 6).

## Discussion

During its clinical development, the 1790GAHB candidate vaccine showed an acceptable safety profile and was shown to induce robust anti-*S. sonnei* LPS serum IgG responses^18,30,31^. Moreover, serum IgG antibodies were bactericidal and 1790GAHB showed ability to boost the response in individuals pre-immunized or naturally exposed to *Shigella*^29,32^. While the vaccine did not show clinical efficacy against shigellosis after challenge with *S. sonnei* in this CHIM trial^33^, the study design allowed us to explore the role of several immunomarkers in predicting the risk of shigellosis.

We found that 1790GAHB-induced low levels of *S. sonnei* LPS-specific fecal sIgA that increased only slightly after the second vaccination, with similar levels observed in the placebo group. Fecal OAg-specific IgA levels have been previously correlated with protection against shigellosis in the case of infection^14^ or post-immunization with live oral *Shigella* vaccines^37,38^, likely as a result of induced mucosal immunity. Thus, at 7 days post-infection with lyophilized *S. sonnei* 53G in a different CHIM trial in adults, LPS-specific fecal IgA responses were higher in volunteers not progressing to shigellosis than those developing shigellosis after challenge. Moreover, fecal IgA responses continued to increase throughout 14 days post-infection only in volunteers not progressing to shigellosis^14^. To our knowledge, there is no other assessment of fecal sIgA responses reported after immunization with parenteral *Shigella* vaccines, as parenteral vaccines are known to induce systemic, rather than mucosal immune responses^8^. As expected, the magnitude of the fecal sIgA response in our study was lower than that reported after the administration of two live oral *S. sonnei* vaccine candidates, WRSs2 and WRSs3^37^. In addition to the parenteral administration of the vaccine, other factors could contribute to the low fecal sIgA responses observed after vaccination with 1790GAHB. For instance, antigen-specific fecal sIgA levels were shown to peak between 10 and 14 days and decrease at 28 days post-vaccination with the live oral WRSs2 and WRSs3 vaccines^37^, therefore the timing of the assessment in our study may not have been optimal to detect an increase in IgA response.

Nevertheless, we found that 1790GAHB was able to induce *S. sonnei* LPS-specific IgG and IgA responses from ASCs with gut-homing receptors. Both IgG and IgA from α4β7+ ASCs peaked at 7 days post-first vaccination with 1790GAHB, with 94% and 91% of vaccine recipients, respectively, showing ≥4-fold increases from baseline, compared with 10% and 3% in the placebo group. The percentage of participants receiving 1790GAHB with ≥4-fold increase in *S. sonnei* LPS specific IgG and IgA levels from α4β7+ ASCs remained higher than in the placebo group at 7 days post-second vaccination. This indicates the ability of the *S. sonnei* GMMA-based vaccine to induce a mucosal response, despite the low fecal sIgA levels observed, as circulating plasmablasts expressing α4β7 integrin are likely homing to the intestinal mucosa. A similar finding was previously reported for another parenteral *Shigella* vaccine, a bioconjugate composed of the O-polysaccharide of *S. flexneri* 2a enzymatically linked to the exotoxin A of *Pseudomonas aeruginosa* (Flexyn2a). Flexyn2a induced α4β7+ IgG and IgA responses that were strongly correlated with each other^39^. Taken together, our results show that parenteral GMMA-based vaccines have the ability to induce mucosal response in addition to systemic antibody responses.

An increase in α4β7− IgG and IgA levels compared to baseline was observed post-vaccination with 1790GAHB in our study, similarly to findings for Flexyn2a^39^. We observed no increase in α4β7− IgG/IgA responses post-challenge in the current trial. In contrast, significant increases in LPS specific α4β7− IgG/IgA responses post-infection with lyophilized *S. sonnei* 53G, in non-immunized adults, were observed in another CHIM trial^14^.

A key factor in the design of vaccines against infectious diseases is determining the phenotype and magnitude of vaccine-induced immune response that confers protection against infection or at least against a severe clinical outcome. Such immunological markers could then be used in assessing VE in clinical trials without having to conduct trials to measure severe clinical outcomes, thus reducing overall cost and time delays. Approval of a vaccine for marketing by health authorities often relies on demonstrating VE based on an accepted surrogate of protection. The application of surrogates of protection for licensure of vaccine candidates has been extensively used for vaccines against meningitis, pneumococcal, and influenza, to mention a few^40^. However, establishing a CoP for many infectious diseases can be extremely complex and to date, there is no accepted CoP for shigellosis. Qin et al. define CoPs as immunological measurements that predict a vaccine’s level of protective efficacy based on differences between the vaccinated and unvaccinated groups. In contrast, CoRs are defined as immunomarkers predicting a clinical endpoint in some populations (i.e., in the context of clinical trials, but without being generalized across settings or populations) and irrespective of the intervention^35^. A CoR is not necessarily a CoP but once identified, it can be validated as a CoP in larger clinical trials, where a vaccine effect is observed. Because we could not demonstrate efficacy against shigellosis following vaccination with 1790GAHB in this CHIM study, a CoP and a threshold predicting VE cannot be identified among the investigated immunomarkers. However, based on the clinical endpoint evaluated (shigellosis), CoRs can be evaluated^34^. We found that at baseline, higher anti-*S. sonnei* LPS-specific serum IgG titers were associated with a reduced risk of shigellosis, while SBA titers were borderline associated with a reduced risk. Our findings for LPS-specific serum IgG further confirm the accumulated data on the role of this immunomarker as a CoP or CoR^13,41,42^. However, there is less evidence in the literature on the use of functional antibody titers as a CoP/CoR for shigellosis. In a previous challenge study, higher baseline SBA titers correlated with resistance to infection post-challenge with *S. sonnei* 53G, although this correlation was less robust than for other assessed immunomarkers, such as LPS-specific serum IgA response^14^. Another CHIM study found a strong association between specific SBA titers against *S. flexneri* 2a and a reduction in disease severity post-challenge^43^. In a different CHIM study evaluating the efficacy of the Flexyn2a bioconjugate vaccine, vaccinees protected against *S. flexneri* 2a shigellosis had higher SBA titers than unprotected vaccine recipients; however, in contrast with the findings from the other CHIM trial, this difference was not statistically significant^39^. Interestingly, in the same study, when assessing correlation with other immunomarkers, bactericidal activity showed the strongest correlation with α4β7+ IgG responses, followed by serum IgG1 responses^39^. We also previously reported a strong correlation between the 1790GAHB elicited SBA titers and anti-*S. sonnei* LPS serum IgG levels in adults from both *Shigella*-endemic^29^ and non-endemic countries^18^, indicating the functionality of antibodies induced by vaccination. The magnitude of antibody responses may play a less significant role in protection than their functionality; for instance, findings from a CHIM study suggest that *Shigella*-specific serum IgG1 may play an important role in protection from shigellosis after parenteral immunization even at relatively low levels elicited post-vaccination^39^, due to their high efficiency in activating the complement cascade^44^. Current recommendations for the prioritization of *Shigella*-specific immunoassays list serum antibody functionality by SBA among first-priority tier analysis^45^. The use of functional antibodies as an immunomarker predictive of protection is well established for other diseases caused by pathogens invading mucosal surfaces, such as pneumococcal and meningococcal infection^19^.

In our study, higher IgA (but not IgG) levels from α4β7+ and α4β7− ASCs post-vaccination, as well as their increase from baseline were associated with reduced risk of shigellosis. However, post-first immunization with Flexyn2a, LPS specific α4β7+/− IgG responses were significantly higher in vaccinees protected versus non-protected against shigellosis post-challenge with virulent *S. flexneri* 2a, whereas no significant differences were observed for α4β7+/− IgA responses^39^. Differences between the elicited response can be due to the nature of the two parenteral vaccines (GMMA-based, Alhydrogel-adjuvanted 1790GAHB versus the bioconjugate unadjuvanted Flexyn2a). In addition, LPS specific α4β7+ IgG and IgA responses have previously been shown to be significantly increased from baseline in volunteers with shigellosis after challenge with *S. sonnei* than those without, indicating a predominantly mucosal immune response after oral challenge with the wild-type organism^14^. In contrast, challenge with *S. flexneri* 2a is believed to lead to a balanced mucosal/systemic response^15^. These differences between serotypes with regard to responses mounted to natural infection may also apply to immunization with serotype-specific vaccines, although the underlying mechanism is most likely different. This corroborates previous observations that the definition of a single immune CoP may not be adequate for *Shigella*, considering the complexities associated with the immune response following infection or vaccination combined with potential differences across diverse serotypes^15^. However, based on our findings, we suggest that future research may focus on the design of vaccines able to elicit mainly anti-*S. sonnei* LPS serum IgG responses, but also to induce high SBA titers and robust LPS-specific α4β7+/− IgG responses. The CoRs identified in our study may also be CoPs, but this needs to be validated in additional clinical trials in which VE is observed. Of note, we also observed differences in the OR of shigellosis between the 1790GAHB and placebo groups, which should not arise in the context of the low VE_CE_ of 1790GAHB observed in the CHIM trial^33^. Therefore, associations between immunomarkers and risk of shigellosis identified in our study are difficult to interpret, and might be at least partly due to the lack of correction for multiplicity. In addition, the CoR analyses were exploratory in nature; therefore, the sample size was not driven by statistical hypothesis and *p*-values were not adjusted by multiple comparisons.

To evaluate whether 1790GAHB elicits antibodies with different specificity able to induce complement-mediated killing, we also tested the SBA of LPS-depleted sera. No residual bactericidal activity against OAg-positive bacteria was detected in sera depleted of anti-LPS antibodies compared to non-depleted sera of 1790GAHB recipients post-vaccination^33^, indicating that 1790GAHB-induced SBA is mediated by anti-LPS antibodies. However, when tested in SBA against a *S. sonnei* OAg-negative strain, anti-GMMA protein antibodies were able to kill the bacteria and a slight increase of SBA titers upon vaccination with 1790GAHB was observed versus no change in participants receiving placebo. This is in line with previous preclinical observations that showed anti-OAg antibodies are the main drivers of SBA against OAg-positive *Shigella* strains, while antibodies not targeting OAg are functional against OAg‑negative bacteria^46,47^.

The anti-*Shigella* LPS-specific IgG avidity analysis suggested an association between *S. sonnei* LPS-specific total IgG avidity and protection from shigellosis. Antibody avidity was similar between 1790GAHB and placebo recipients regardless of the challenge outcome, suggesting that this association is not impacted by vaccination and that this immunomarker may be a suitable CoP. When grouped by both treatment and challenge outcome, the median avidity index for participants who did not develop shigellosis post-challenge with *S. sonnei* was higher than that for participants developing shigellosis in the placebo group. Only a trend for higher antibody avidity was observed in the vaccine group. However, these results require confirmation in a larger population and should be interpreted with caution due to the relatively small number of samples tested, which also varied across timepoints.

The *S. sonnei* LPS serum IgG protection threshold of 1:1600 proposed by Cohen et al. in young Israeli adults^13^ was not validated in US adults in this CHIM study. In our study, the VE_IM_ and VE_CE_ point estimates were not comparable (39.4% versus −9.4%), although a slight overlap of the 90% CI was observed. To formally assess the equivalence of VE_IM_ and VE_CE_, a large population and a priori-defined equivalence margins are required and this was not possible with the small sample size of a CHIM study^33^. Therefore, although our data confirmed the role of anti-*S. sonnei* LPS serum IgG as a CoR for shigellosis, we could not provide evidence for its role as a CoP, nor that the 1:1600 titer threshold can be used to predict efficacy. There were also other factors that were hindering this comparison. First, the assays used in the two studies were different and required a conversion that may have led to additional uncertainty and variability of the data. Second, VE is known to differ with setting^48^, and the Cohen et al. study was conducted in field settings, with shigellosis cases being collected 71–155 days post-vaccination^13^, while we tested the CoP in a CHIM model and collected shigellosis cases during 29–36 days post-vaccination. Finally, the confidence limits around the VE_IM_ estimate did not account for variability around threshold, therefore the real CIs might be larger than those reported.

The results of this CHIM study supported the design of an improved version of the four-component GMMA vaccine, which was formulated with 10-fold higher OAg amount for *S. sonnei* and three additional serotypes (*S. flexneri* 1b, 2a and 3a)^27^. This candidate vaccine is now being tested in adults 18–50 years of age from non-endemic *Shigella* countries, as a first stage of a phase 1/2 study (NCT05073003) before progressing to investigations in endemic settings.

Overall study limitations have been previously discussed in detail^33^ and include the low sample size and the fact that there was no assessment of *S. sonnei* LPS-specific serum IgA or IgG isotyping. In addition to these and the limitations discussed for the CoR analysis, the study had all limitations inherent to a CHIM^48^.

In conclusion, although these analyses cannot validate a CoP that predicts VE, they indicated immunological measures which are associated with reduced risk of shigellosis, and further confirm the critical role of LPS in protection. We were unable to establish or confirm threshold levels that can predict protection against shigellosis, which would have facilitated the development and evaluation of *Shigella* vaccine candidates. However, we confirmed serum anti-LPS IgG and identified bactericidal activity as potential CoRs for shigellosis.

## Methods

### Study design and objectives

We conducted a single-center, observer-blind, randomized, placebo-controlled, phase 2b CHIM study between August 2018 and November 2019 at the Cincinnati Children’s Hospital Medical Center (CCHMC), Ohio, USA. The study design was previously described in detail^33^. Briefly, participants aged 18–50 years were randomized (1:1) to receive two doses of either 1790GAHB (0.5 mL dose containing 1.5/25 μg of OAg/protein) or placebo (0.5 mL of Alhydrogel in tris-buffered saline [0.7 mg Al^3+^/mL]), 28 days apart and were challenged on D57 with *S. sonnei* strain 53G.

The study was conducted in accordance with all applicable regulatory requirements, the Good Clinical Practice guidelines, and the Declaration of Helsinki. The protocol and study-related documents were approved by the CCHMC institutional review board, and all participants provided written informed consent. The trial is registered on ClinicalTrials.gov (NCT03527173), and the full study protocol is available at www.gsk-studyregister.com (study ID 205626).

The results of the primary and secondary objectives have previously been reported, including serum anti-S*. sonnei* LPS IgG levels and SBA titers^33^. Here, we present the results of tertiary and exploratory objectives that evaluated *S. sonnei* specific sIgA in stool, as well as *S. sonnei* LPS IgA/IgG specific α4β7+/α4β7− ASC plasmablast response pre- and post-vaccination, as indicated in Fig. 2. We also assessed the association with shigellosis for all evaluated immunomarkers, including those previously reported^33^, and investigated their role as CoRs. Furthermore, in post-hoc analyses, we evaluated SBA against OAg-positive and OAg-negative *Shigella* strains after anti-LPS antibody depletion and anti-*S. sonnei* LPS-specific total IgG avidity (Fig. 2). In addition, we explored the role of the CoP proposed by Cohen et al. in young Israeli adults^13^ in the population of US adults from the CHIM trial^33^.

Blood samples for antibody response and evaluation of SBA (~20 mL) and for PBMC isolation (α4β7+/α4β7− plasmablasts response) (~50 mL) and stool samples for the detection of fecal sIgA were collected as indicated in Fig. 2.

*S. sonnei* LPS fecal sIgA were determined by an ELISA at CCHMC. Stool (2.5–3.0 g) was mixed well with extraction buffer and then centrifuged (20,000 revolutions/minute) for 30 min at 4 °C. The supernatant was collected and transferred into pre-labeled cryovials. Antigen-specific LPS sIgA and total IgA were determined by ELISA. Total IgA were expressed as mg/mL and the LPS-specific sIgA as titer. Results were reported as LPS activity (titers/mg/mL), defined as LPS sIgA divided by total IgA. The lower limit of quantification (LLOQ) for the LPS sIgA was 10. The assay was performed CCHMC Laboratories for Specialized Clinical Studies (Cincinnati, Ohio, US).

*S. sonnei* LPS-specific IgA/IgG from α4β7+/α4β7− ASCs were assessed from PBMC. Cells were separated into α4β7+ and α4β7− populations. Both cell populations were cultured in vitro to collect antibodies in lymphocyte supernatant (ALS). The ALS samples were assayed using ELISA as previously described^14^ to determine *S. sonnei* LPS-specific endpoint titers (expressed as titer/5*10^6^ cells). The assay was performed at the Walter Reed Army Institute of Research (Silver Spring, Maryland, US).

*S. sonnei* bactericidal titers after LPS specific antibodies depletion were determined using a luminescent serum bactericidal assay^33,49^, with LLOQs of 33 and 10 of inhibition concentration (IC)50 for the *S. sonnei* OAg-positive and OAg-negative strains, respectively. Prior to the assay, LPS antibody depletion was performed by overnight incubation with homologous competitor *S. sonnei* LPS (at a concentration of 50 µg/mL) and confirmed by ELISA using *S. sonnei* LPS as plate coating antigen (at a concentration of 0.5 µg/mL in phosphate buffer saline [PBS]). To prove a minimal reduction of anti-GMMA protein antibodies, we also performed ELISA using *S. sonnei* OAg-negative GMMA as plate coating antigen (at a concentration of 1 µg/mL in PBS) of samples pre- and post-LPS antibodies depletion. Effective depletion was confirmed with a reduction of EU/mL on LPS coating >80% and <30% on GMMA OAg-negative coating. These assays were performed at GVGH.

The anti-*S. sonnei* LPS-specific total IgG avidity was determined by ELISA^33^ in the presence of chaotropic agent. The avidity index was expressed as the ratio of IgG concentration (EU/mL) after treatment with 0.5 M ammonium thiocyanate, compared to the value for the same sample without chaotropic treatment. The LLOQ was 9.9 EU/mL. The assay was performed at GVGH.

### Statistical analysis

All statistical analyses were carried out using Statistical Analysis Systems 9.4.

Sample size considerations have previously been described^33^. All immunogenicity analyses (except anti-*S. sonnei* LPS specific total IgG avidity index) used descriptive statistics and were conducted in the per-protocol set for immunogenicity comprising all participants with available data in the full analysis set who correctly received the vaccine/placebo, had no major protocol deviation, and had immunogenicity data at the relevant timepoint. Anti-*S. sonnei* LPS-specific total IgG avidity index was assessed in a subset of 26 participants in each of the 1790GAHB and the placebo groups.

For Analysis of immunogenicity, for each group, median, median difference, and percentage of participants with ≥4-fold increase post-vaccination in S. sonnei LPS specific fecal sIgA activity compared to baseline and pre-challenge were calculated with two-sided 95% CIs. LPS-specific sIgA activity corresponding to participants with titers below the LLOQ was set to zero. For analysis purposes, to perform log_10_ transformation, zero values were recoded to the smallest non-zero value in the dataset, divided by 10 before log_10_ transformation.

Median, median difference, and the number and percentage of participants with a ≥4-fold increase post-vaccination in antibody titers against *S. sonnei* LPS specific IgA/IgG from α4β7+ and α4β7− ASC plasmablasts were calculated with 95% CIs.

For the evaluation of *S. sonnei* bactericidal titers after LPS antibody depletion, geometric mean titers, geometric mean ratios, and the percentage of participants with a ≥4-fold increase post-vaccination compared to baseline (or a 4-fold increase compared to LLOQ, if the participant had SBA titers <LLOQ) were calculated with 2-sided 95% CIs. Titers below the LLOQ were set to half that limit for the purpose of the analysis.

Mean anti-*S. sonnei* LPS-specific total IgG avidity indices with 95% CIs were computed. For analysis purposes, when serum concentration after chaotropic treatment was below the LLOQ, its value was replaced by half of the LLOQ (4.95) and then the avidity index was calculated; values > 1 were replaced by 1.

Analyses of correlates of risk for shigellosis were conducted in the per-protocol set for efficacy^33^, using univariate logistic regression with shigellosis as dependent variable (present versus absent) and the immunomarker as continuous independent variable after log_10_ transformation. ORs were calculated with Wald 95% CIs and *p*-values; the Firth method^50^ was used for CIs in case of quasi-complete separation of data. The ORs quantify the ratio between odds of shigellosis for a 10-fold increase in immunomarker (original non-transformed value) between different participants at the same timepoint or between baseline and post-vaccination timepoint within the same participant; ORs <1 indicate that higher values are associated with lower risk of shigellosis. Immunomarkers with *p* < 0.05 from the logistic regression using baseline pooled data were considered CoRs. *P*-values from all the other remaining logistic regressions were calculated to further explore the association between immunomarkers and shigellosis. Due to the descriptive nature of all analyses, *p*-values and CIs were not adjusted for multiplicity.

Exploration of correlate of protection against Shigella was done by calculating VE_IM_ on the per-protocol efficacy set from the US adults in the CHIM study using a model proposed by Siber et al.^51^ as:

$${{VE}}_{{IM}}=1-\frac{{Proportion}\,{of}1790{GAHB}\,{vaccinees}\,{with}\,S.{sonnei}\,{IgG}\,{below}\,{protection}\,{threshold}}{{Proportion}\,{of}\,{placebo}\,{recipients}\,{with}\,S.{sonnei}\,{IgG}\,{below}\,{protection}{threshold}}$$

In the above formula, the titer of 1:1600 measured using the TAU assay was used as the protection threshold, as identified by Cohen et al.^13^. To allow comparability between VE_IM_ and VE_CE_, the CIs were calculated with the same method, i.e., 90% 2-sided exact unconditional CIs with the Miettinen–Nurminen method^52^. This method is expected to underestimate variance of VE_IM_ as it makes the strong assumption that the threshold has no variability, however it was not possible to use the bootstrap method, as we had no access to original data.

In a commutability study, the 1:1600 titer threshold in the TAU assay was shown to correspond to 396 EU/mL in the in-house ELISA currently used by the GVGH. These conversions were performed to allow between-study comparisons. As in the current GVGH ELISA assay the standard serum has been recalibrated with respect to the one used in the CHIM^33^, ELISA measurements obtained in the original CHIM study were multiplied by 0.45 to correspond to the in-house ELISA currently used by the GVGH.

### Reporting summary

Further information on research design is available in the Nature Research Reporting Summary linked to this article.

### Supplementary information

Additional details

REPORTING SUMMARY

## Data Availability

Anonymized individual participant data and study documents can be provided upon request from www.clinicalstudydatarequest.com.
