# Supplementary material for: Putative correlates of protection against shigellosis assessing immunomarkers across responses to S. sonnei investigational vaccine
Source: NPJ Vaccines. 2024 Mar 8;9:56. doi: 10.1038/s41541-024-00822-2 (PMC10923941; doi:10.1038/s41541-024-00822-2)
Supplement: Supplementary file 1 — Additional details [file 41541_2024_822_MOESM1_ESM.pdf]

Supplementary material

Putative correlates of protection against shigellosis: assessing immunomarkers across immune responses to *S. sonnei* investigational vaccine 1790GAHB

Valentino Conti, Omar Rossi, Kristen A. Clarkson, Francesca Mancini, Usman N. Nakakana, Eleanna Sarakinou, Andrea Callegaro, Pietro Ferruzzi, Alessandra Acquaviva, Ashwani Kumar Arora, Elisa Marchetti, Francesca Necchi, Robert W. Frenck Jr, Laura B. Martin, Robert W. Kaminski, Audino Podda, Francesca Micoli

Contents

Supplementary Figure 1. Percentage of participants with ≥4-fold increase in levels of *S. sonnei* LPS fecal sIgA (a) and *S. sonnei* LPS specific IgG/IgA levels from α4β7+ and α4β7- ASCs (b-e), by outcome (no shigellosis versus shigellosis) post-challenge dose (per-protocol efficacy set) 2

Supplementary Table 1. Summary of demography and baseline characteristics (per-protocol immunogenicity set).......... 3

Supplementary Table 2. Summary of immunogenicity results, overall 4

Supplementary Table 3. Correlate of risk analysis across all assays (per-protocol set for efficacy).......... 8

Supplementary Figure 1. Percentage of participants with ≥4-fold increase in levels of *S. sonnei* LPS fecal sIgA (a) and *S. sonnei* LPS specific IgG/IgA levels from α4β7+ and α4β7- ASCs (b-e), by outcome (no shigellosis versus shigellosis) post-challenge dose (per-protocol efficacy set)

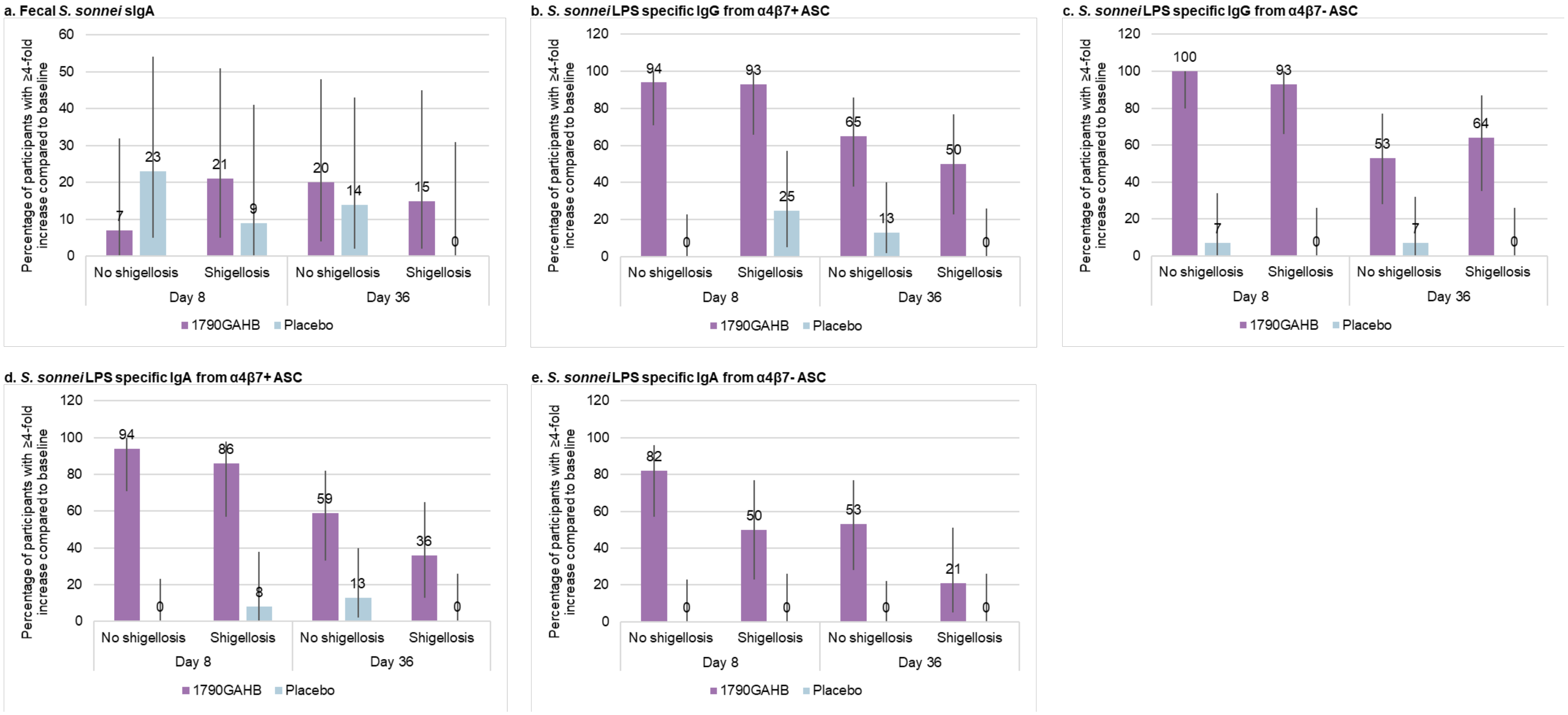

sIgA, secretory immunoglobulin A; LPS, lipopolysaccharide; Ig, immunoglobulin; ASC, antibody-secreting cell.

Note: Error bars represent 95% confidence intervals.

Supplementary Table 1. Summary of demography and baseline characteristics (per-protocol immunogenicity set)

	1790GAHB group (N=34)	Placebo group (N=31)	Total (N=65)
Age at first vaccination, years			
Mean ± standard deviation	36.1±9.8	34.5±8.7	35.3±9.3
Median (minimum; maximum)	37.0 (19; 50)	31.0 (19; 49)	33.0 (19; 50)
Male sex, n (%)	21 (61.8)	16 (51.6)	37 (56.9)
Hispanic or Latino ethnicity, n (%)	1 (2.9)	1 (3.2)	2 (3.1)
Race, n (%)			
Black or African American	22 (64.7)	26 (83.9)	48 (73.8)
White	10 (29.4)	5 (16.1)	15 (23.1)
Other	2 (5.9)	0 (0.0)	2 (3.1)

N, number of participants; n (%), number (percentage) of participants in each category.

Supplementary Table 2. Summary of immunogenicity results, overall

	1790GAHB group		Placebo group	
	N	Value	N	Value
***S. sonnei* fecal sIgA**				
D1, median (min; max)	30	1.45 (1.45; 348.60)	25	1.45 (1.45; 345.45)
D8, median (min; max)	30	1.45 (1.45; 3682.56)	24	1.45 (1.45; 424.94)
% (95% CI) of participants with ≥4-fold increase from D1	30	13% (4–31)	24	17% (5–37)
Difference from D1, median (min; max)		0.00 (-117.56; 3681.11)		0.00 (-314.59; 423.49)
D36, median (min; max)	29	1.45 (1.45; 12337.68)	24	1.45 (1.45; 385.56)
% (95% CI) of participants with ≥4-fold increase from D1	29	17% (6–36)	24	8% (1–27)
Difference from D1, median (min; max)		0.00 (-280.04; 11989.08)		0.00 (-204.81; 384.11)
D64, median (min; max)	27	35.04 (1.45; 3899.25)	24	34.51 (1.45; 267.24)
% (95% CI) of participants with ≥4-fold increase from D1	27	48% (29–68)	24	54% (33–74)
Difference from D1, median (min; max)		19.46 (-87.44; 3550.65)		20.14 (-314.59; 265.79)
% (95% CI) of participants with ≥4-fold increase from D36	26	42% (23–63)	23	48% (27–69)
Difference from D57, median (min; max)		6.43 (-8438.43; 325.01)		28.91 (-333.05; 241.93)
***S. sonnei* LPS specific IgG from α4β7+ ASC**				
D1, median (min; max)	33	1.00 (1.00; 9.00)	31	1.00 (1.00; 256.00)
D8, median (min; max)	33	128.00 (2.00; 4096.00)	29	1.00 (1.00; 256.00)
% (95% CI) of participants with ≥4-fold increase from D1	33	94% (80–99)	29	10% (2–27)
Difference from D1, median (min; max)		127.00 (1.00; 4095.00)		0.00 (-247.00; 255.00)
D36, median (min; max)	33	4.00 (1.00; 256.00)	30	1.00 (1.00; 16.00)
% (95% CI) of participants with ≥4-fold increase from D1	33	61% (42–77)	30	7% (1–22)
Difference from D1, median (min; max)		3.00 (-2.00; 255.00)		0.00 (-255.00; 15.00)
D64, median (min; max)	32	3.00 (1.00; 17467.00)	29	8.00 (1.00; 4452.00)
% (95% CI) of participants with ≥4-fold increase from D1	32	47% (29–65)	29	52% (33–71)
Difference from D1, median (min; max)		2.00 (-1.00; 17466.00)		7.00 (-2.00; 4451.00)

	1790GAHB group		Placebo group	
	N	Value	N	Value
% (95% CI) of participants with ≥4-fold increase from D36	32	34% (19–53)	28	50% (31–69)
Difference from D57, median (min; max)		0.00 (-127.00; 17435.00)		7.00 (-14.00; 4451.00)
***S. sonnei* LPS specific IgG from α4β7- ASC**				
D1, median (min; max)	33	1.00 (1.00; 4.00)	31	1.00 (1.00; 128.00)
D8, median (min; max)	33	64.00 (1.00; 2048.00)	29	1.00 (1.00; 4.00)
% (95% CI) of participants with ≥4-fold increase from D1	33	97% (84–100)	29	3% (0–18)
Difference from D1, median (min; max)		63.00 (0.00; 2047.00)		0.00 (-126.00; 3.00)
D36, median (min; max)	33	4.00 (1.00; 128.00)	30	1.00 (1.00; 32.00)
% (95% CI) of participants with ≥4-fold increase from D1	33	61% (42–77)	30	3% (0–17)
Difference from D1, median (min; max)		3.00 (-1.00; 127.00)		0.00 (-127.00; 31.00)
D64, median (min; max)	32	1.50 (1.00; 16.00)	29	1.00 (1.00; 256.00)
% (95% CI) of participants with ≥4-fold increase from D1	32	22% (9–40)	29	24% (10–44)
Difference from D57, median (min; max)		0.00 (0.00; 15.00)		0.00 (-2.00; 255.00)
% (95% CI) of participants with ≥4-fold increase from D36	32	6% (1–21)	28	29% (13–49)
Difference from D57, median (min; max)		-2.00 (-124.00; 14.00)		0.00 (-31.00; 255.00)
***S. sonnei* LPS specific IgA from α4β7+ ASC**				
D1, median (min; max)	33	1.00 (1.00; 9.00)	31	1.00 (1.00; 256.00)
D8, median (min; max)	33	128.00 (2.00; 2048.00)	29	1.00 (1.00; 6.00)
% (95% CI) of participants with ≥4-fold increase from D1	33	91% (76–98)	29	3% (0–18)
Difference from D1, median (min; max)		127.00 (1.00; 2047.00)		0.00 (-254.00; 3.00)
D36, median (min; max)	33	4.00 (1.00; 256.00)	30	1.00 (1.00; 8.00)
% (95% CI) of participants with ≥4-fold increase from D1	33	52% (34–69)	30	7% (1–22)
Difference from D1, median (min; max)		3.00 (-2.00; 255.00)		0.00 (-255.00; 7.00)
D64, median (min; max)	32	3.00 (1.00; 512.00)	29	8.00 (1.00; 2048.00)
% (95% CI) of participants with ≥4-fold increase from D1	32	47% (29–65)	29	52% (33–71)
Difference from D1, median (min; max)		1.00 (-6.00; 511.00)		7.00 (0.00; 2047.00)

	1790GAHB group		Placebo group	
	N	Value	N	Value
% (95% CI) of participants with ≥4-fold increase from D36	32	34% (19–53)	28	54% (34–72)
Difference from D57, median (min; max)		0.00 (-254.00; 511.00)		7.00 (-3.00; 2047.00)
***S. sonnei* LPS specific IgA from α4β7- ASC**				
D1, median (min; max)	33	1.00 (1.00; 4.00)	31	1.00 (1.00; 32.00)
D8, median (min; max)	33	15.00 (1.00; 512.00)	29	1.00 (1.00; 4.00)
% (95% CI) of participants with ≥4-fold increase from D1	33	70% (51–84)	29	3% (0–18)
Difference from D1, median (min; max)		11.00 (0.00; 511.00)		0.00 (-31.00; 3.00)
D36, median (min; max)	33	2.00 (1.00; 128.00)	30	1.00 (1.00; 2.00)
% (95% CI) of participants with ≥4-fold increase from D1	33	39% (23–58)	30	0% (0–12)
Difference from D1, median (min; max)		1.00 (-1.00; 127.00)		0.00 (-31.00; 1.00)
D64, median (min; max)	32	1.00 (1.00; 32.00)	29	1.00 (1.00; 64.00)
% (95% CI) of participants with ≥4-fold increase from D1	32	6% (1–21)	29	14% (4–32)
Difference from D1, median (min; max)		0.00 (-1.00; 31.00)		0.00 (-28.00; 63.00)
% (95% CI) of participants with ≥4-fold increase from D36	32	3% (0–16)	28	18% (6–37)
Difference from D57, median (min; max)		0.00 (-127.00; 28.00)		0.00 (-1.00; 63.00)
**Bactericidal titers against OAg-positive *Shigella* strains using sera depleted of LPS**				
D1, GMT (95% CI)	34	16.50 (16.50–16.50)	31	16.50 (16.50–16.50)
D29, GMT (95% CI)	34	16.50 (16.50–16.50)	31	16.50 (16.50–16.50)
% (95% CI) of participants with ≥4-fold increase from D1		0% (0–10)		0% (0–11)
GMR (95% CI)		1 (1–1)		1 (1–1)
D57, GMT	34	16.50 (16.50–16.50)	31	16.50 (16.50–16.50)
% (95% CI) of participants with ≥4-fold increase from D1		0% (0–10)		0% (0–11)
GMR		1 (1–1)		1 (1–1)
**Bactericidal titers against OAg-negative *Shigella* strains using sera depleted of LPS**				
D1, GMT (95% CI)	34	355.11 (263.98–477.71)	31	316.21 (239.55–417.39)
D29, GMT (95% CI)		481.79 (366.49–633.37)		312.65 (236.42–413.45)

	1790GAHB group		Placebo group	
	N	Value	N	Value
% (95% CI) of participants with ≥4-fold increase from D1	34	6% (1–20)	31	0% (0–11)
GMR (95% CI)		1.36 (1.11–1.66)		0.99 (0.90–1.09)
D57, GMT(95% CI)	34	503.38 (362.60–698.81)	31	291.78 (217.64–391.17)
% (95% CI) of participants with ≥4-fold increase from D1		9% (2–24)		0% (0–11)
GMR (95% CI)		1.42 (1.15–1.75)		0.92 (0.84–1.01)

N, number of participants with available results at each timepoint; D, day; CI, confidence interval; sIgA, secretory immunoglobulin A; LPS, lipopolysaccharide; Ig, immunoglobulin; ASC, antibody-secreting cell; OAg, O antigen; GMT, geometric mean titer; GMR, geometric mean ratio.

Supplementary Table 3. Correlate of risk analysis across all assays (per-protocol set for efficacy)

	1790GAHB group			Placebo group			Overall		
	N	OR (95% CI)	p-value	N	OR (95% CI)	p-value	N	OR (95% CI)	p-value
***S. sonnei* LPS IgG**									
Day 1	32	0.172 (0.037–0.786)	0.023	28	0.174 (0.028–1.099)	0.063	60	0.175 (0.055–0.553)	0.003
Day 57	32	0.326 (0.095–1.121)	0.075	28	0.187 (0.028–1.257)	0.085			
Change from Day 1 to Day 57	32	1.680 (0.278–10.149)	0.572	28	219.4 (0.070–>999.9)	0.189			
***S. sonnei* SBA**									
Day 1	32	0.036 (<0.001–0.472)*	NA**	28	0.139 (0.002–8.265)	0.344	60	0.023 (<0.001–0.986)	0.049
Day 57	32	0.168 (0.042–0.673)	0.012	28	0.235 (0.024–2.292)	0.213			
Change from Day 1 to Day 57	32	0.564 (0.175–1.819)	0.338	28	0.426 (0.030–6.049)	0.528			
***S. sonnei* fecal sIgA**									
Day 1	29	0.790 (0.342–1.825)	0.581	25	0.454 (0.152–1.351)	0.156	54	0.639 (0.337–1.214)	0.171
Day 36	31	0.707 (0.318–1.573)	0.395	27	0.219 (0.061–0.785)	0.020			
Change from Day 1 to Day 36	28	0.776 (0.353–1.704)	0.528	24	0.287 (0.021–3.877)	0.347			
***S. sonnei* LPS specific IgG from α4β7+ ASC**									
Day 1	31	<0.001 (<0.001–1.102)*	NA**	28	3.659 (0.385–34.765)	0.259	59	1.407 (0.347–5.704)	0.633
Day 36	31	0.572 (0.196–1.670)	0.307	27	0.391 (0.032–4.861)	0.466			
Change from Day 1 to Day 36	31	0.759 (0.272–2.121)	0.599	27	0.082 (0.002–3.104)	0.177			
***S. sonnei* LPS specific IgG from α4β7- ASC**									
Day 1	31	1.601 (0.006–418.73)	0.868	28	2.214 (0.272–18.009)	0.457	59	2.049 (0.301–13.952)	0.464
Day 36	31	0.704 (0.213–2.324)	0.565	27	0.253 (0.006–11.385)	0.479			
Change from Day 1 to Day 36	31	0.701 (0.218–2.225)	0.551	27	0.238 (0.015–3.754)	0.308			
***S. sonnei* LPS specific IgA from α4β7+ ASC**									
Day 1	31	0.248 (0.010–6.342)	0.399	28	2.078 (0.373–11.576)	0.404	59	1.259 (0.339–4.681)	0.731
Day 36	31	0.176 (0.037–0.834)	0.029	27	0.365 (0.015–9.141)	0.540			
Change from Day 1 to Day 36	31	0.265 (0.065–1.072)	0.063	27	0.263 (0.024–2.868)	0.273			
***S. sonnei* LPS specific IgA from α4β7- ASC**									
Day 1	31	0.059 (<0.001–11.423)*	NA**	28	1.698 (0.151–19.152)	0.668	59	0.910 (0.099–8.365)	0.934
Day 36	31	0.137 (0.024–0.794)	0.027	27	0.006 (<0.001–38.614)*	NA**			
Change from Day 1 to Day 36	31	0.177 (0.034–0.915)	0.039	27	0.056 (<0.001–143.88)	0.471			
***S. sonnei* LPS specific total IgG avidity**									
Day 29	26	0.101 (0.002–6.594)	0.2826	26	<0.001 (<0.001–0.195)	0.0162			
Day 57	26	0.015 (<0.001–1.747)	0.0835	26	<0.001 (<0.001–0.082)	0.0121			

N, number of participants with available results; OR, odds ratio; CI, Wald confidence interval; LPS, lipopolysaccharide; Ig, immunoglobulin; SBA, serum bactericidal activity; NA, not applicable; sIgA, secretory immunoglobulin A; ASC, antibody-secreting cell.

Notes: OR values <1 indicate that higher values were associated with lower risk of shigellosis. At baseline, data for the 1790GAHB and Placebo groups were pooled.

*Penalized likelihood estimate CI obtained by Firth method;

**p-values can be inaccurate, so inference should be focused on the CIs.
