# Supplementary material for: Putative correlates of protection against shigellosis assessing immunomarkers across responses to S. sonnei investigational vaccine
Source: NPJ Vaccines. 2024 Mar 8;9:56. doi: 10.1038/s41541-024-00822-2 (PMC10923941; doi:10.1038/s41541-024-00822-2)
Supplement: Supplementary file 2 — REPORTING SUMMARY [file 41541_2024_822_MOESM2_ESM.pdf]

nature portfolio

Corresponding author(s): DBPR NPJVACCINES-03211

Last updated by author(s): Aug 29, 2023

Reporting Summary

Nature Portfolio wishes to improve the reproducibility of the work that we publish. This form provides structure for consistency and transparency in reporting. For further information on Nature Portfolio policies, see our Editorial Policies and the Editorial Policy Checklist.

Statistics

For all statistical analyses, confirm that the following items are present in the figure legend, table legend, main text, or Methods section.

n/a	Confirmed	
☐	☒	The exact sample size (*n*) for each experimental group/condition, given as a discrete number and unit of measurement
☐	☒	A statement on whether measurements were taken from distinct samples or whether the same sample was measured repeatedly
☐	☒	The statistical test(s) used AND whether they are one- or two-sided<br>*Only common tests should be described solely by name; describe more complex techniques in the Methods section.*
☒	☐	A description of all covariates tested
☒	☐	A description of any assumptions or corrections, such as tests of normality and adjustment for multiple comparisons
☐	☒	A full description of the statistical parameters including central tendency (e.g. means) or other basic estimates (e.g. regression coefficient) AND variation (e.g. standard deviation) or associated estimates of uncertainty (e.g. confidence intervals)
☒	☐	For null hypothesis testing, the test statistic (e.g. *F*, *t*, *r*) with confidence intervals, effect sizes, degrees of freedom and *P* value noted<br>*Give P values as exact values whenever suitable.*
☒	☐	For Bayesian analysis, information on the choice of priors and Markov chain Monte Carlo settings
☒	☐	For hierarchical and complex designs, identification of the appropriate level for tests and full reporting of outcomes
☒	☐	Estimates of effect sizes (e.g. Cohen's *d*, Pearson's *r*), indicating how they were calculated

Our web collection on statistics for biologists contains articles on many of the points above.

Software and code

Policy information about availability of computer code

Data collection	Data were collected using Central Designer.
Data analysis	All statistical analyses were carried out using Statistical Analysis Systems v9.4.

For manuscripts utilizing custom algorithms or software that are central to the research but not yet described in published literature, software must be made available to editors and reviewers. We strongly encourage code deposition in a community repository (e.g. GitHub). See the Nature Portfolio guidelines for submitting code & software for further information.

Data

Policy information about availability of data

All manuscripts must include a data availability statement. This statement should provide the following information, where applicable:

- Accession codes, unique identifiers, or web links for publicly available datasets
- A description of any restrictions on data availability
- For clinical datasets or third party data, please ensure that the statement adheres to our policy

Anonymized individual participant data and study documents can be provided upon request from www.clinicalstudydatarequest.com.

Research involving human participants, their data, or biological material

Policy information about studies with human participants or human data. See also policy information about sex, gender (identity/presentation), and sexual orientation and race, ethnicity and racism.

Reporting on sex and gender	The study enrolled both male (n=37) and female (n=28) participants, sex was based on self-reporting. The number of participants of each sex included in the analyses is reported in Table S1 in the Supplementary material. Disaggregated sex and gender data were not collected. Sex- and gender-based analyses were not performed due to the relatively low sample size.
Reporting on race, ethnicity, or other socially relevant groupings	The number of participants of Hispanic or Latino ethnicity, as well as participants of each race is reported in Table S1. Ethnicity and race were assigned based on the 1997 US Office of Management and Budget (OMB) standards on race and ethnicity. No ethnicity- or race-based analyses were performed due to the relatively low sample size.
Population characteristics	Population characteristics are reported in Table S1 in the Supplementary material; more details are available in the original publication, reference 33 in the manuscript (Frenck, R. W., Jr et al. Efficacy, safety, and immunogenicity of the Shigella sonnei 1790GAHB GMMA candidate vaccine: Results from a phase 2b randomized, placebo-controlled challenge study in adults. EClinicalMedicine 39, 101076 (2021). https://doi.org:10.1016/j.eclinm.2021.101076)
Recruitment	Recruitment was done at the Cincinnati Children's Hospital Medical Center (Cincinnati, Ohio, United States). Information on recruiting and inclusion/exclusion criteria is presented in the Methods section (Study design and objectives) and in more detail in reference 33 cited in the manuscript, Frenck, R. W., Jr et al. Efficacy, safety, and immunogenicity of the Shigella sonnei 1790GAHB GMMA candidate vaccine: Results from a phase 2b randomized, placebo-controlled challenge study in adults. EClinicalMedicine 39, 101076 (2021). https://doi.org:10.1016/j.eclinm.2021.101076
Ethics oversight	The study protocol, any amendments, the informed consent, and other information that required pre-approval were reviewed and approved by the Institutional Review Board of the Cincinnati Children's Hospital Medical Center (Cincinnati, Ohio, United States). This information is reported in the Methods section (Study design and objectives).

Note that full information on the approval of the study protocol must also be provided in the manuscript.

Field-specific reporting

Please select the one below that is the best fit for your research. If you are not sure, read the appropriate sections before making your selection.

☒ Life sciences ☐ Behavioural & social sciences ☐ Ecological, evolutionary & environmental sciences

For a reference copy of the document with all sections, see nature.com/documents/nr-reporting-summary-flat.pdf

Life sciences study design

All studies must disclose on these points even when the disclosure is negative.

Sample size	Sample size considerations have previously been described in the primary paper of the study, reference 33 cited in the manuscript (Frenck, R. W., Jr et al. Efficacy, safety, and immunogenicity of the Shigella sonnei 1790GAHB GMMA candidate vaccine: Results from a phase 2b randomized, placebo-controlled challenge study in adults. EClinicalMedicine 39, 101076 (2021). https://doi.org:10.1016/j.eclinm.2021.101076): Based on the percentage of participants with a seroresponse (defined as an increase in post-vaccination anti-S. sonnei LPS serum IgG concentrations of at least 50% for participants with pre-vaccination levels >50 enzyme linked immunosorbent assay (ELISA) units (EU), or an increase of at least 25 EU for participants with pre-vaccination levels =<50 EU) observed in previous studies, a VE of 70% was assumed. Based on the results obtained by CCHMC in volunteers challenged with 1500 CFU, an attack rate (AR) for the primary case definition of 58% in the placebo group was assumed. A total number of 21 confirmed cases was needed to demonstrate that the LL of the two-sided 90% CI for the VE was above 0% with 80% power (by one proportion power analysis, one-sided test, one-sided alpha = 5%). Considering an AR of 58% in the placebo group and a percentage of non-evaluable participants of 22%, a sample size of approximately 72 individuals (36 per group / 18 per cohort) was estimated to reach the 21 shigellosis cases.
Data exclusions	All immunogenicity analyses (except anti-S. sonnei LPS specific total IgG avidity index) used descriptive statistics and were conducted in the per-protocol set for immunogenicity comprising all participants with available data in the full analysis set who correctly received the vaccine/placebo, had no major protocol deviation, and had immunogenicity data at the relevant timepoint. Anti-S. sonnei LPS specific total IgG avidity index was assessed in a subset of 26 participants in each of the 1790GAHB and the placebo groups. This information is available in the Methods section (Statistical analysis).
Replication	All assays were set up and fit-for-purpose was characterized before running the assay, and all experiments were conducted according to standard operating procedures. No data meeting the quality control criteria of the operating procedure governing the assay were excluded.
Randomization	Participants were randomized (1:1) to receive 2 doses of either 1790GAHB (1790GAHB group) or placebo (placebo group), using the GSK System Built for Internet Randomization (SBIR). These details are available in the study protocol available at www.gsk-studyregister.com (study ID 205626), as mentioned in the Methods section (Study design and objectives).
Blinding	Data were collected in an observer-blind manner: the vaccine/placebo recipients and those responsible for the evaluation of any study endpoint (e.g. safety, reactogenicity, immunogenicity and efficacy) were unaware of which group the subject belonged (vaccine or placebo). To do so, vaccine/placebo administration was done by authorized unblinded medical personnel who did not participate in any of the study clinical evaluation assays. The study vaccine and placebo were prepared by an unblinded pharmacist. The laboratories in charge of the

laboratory testing were blinded to the treatment, participant and visit number. Codes were used to link the participant and study (without any link to the treatment attributed to the participant) to each sample. These details are available in the study protocol available at www.gsk-studyregister.com (study ID 205626), as mentioned in the Methods section (Study design and objectives).

Reporting for specific materials, systems and methods

We require information from authors about some types of materials, experimental systems and methods used in many studies. Here, indicate whether each material, system or method listed is relevant to your study. If you are not sure if a list item applies to your research, read the appropriate section before selecting a response.

Materials & experimental systems

n/a	Involved in the study	
☐	☒	Antibodies
☒	☐	Eukaryotic cell lines
☒	☐	Palaeontology and archaeology
☒	☐	Animals and other organisms
☐	☒	Clinical data
☒	☐	Dual use research of concern
☒	☐	Plants

Methods

n/a	Involved in the study	
☒	☐	ChIP-seq
☒	☐	Flow cytometry
☒	☐	MRI-based neuroimaging

Antibodies

Antibodies used

Antibodies from the serum samples elicited to S. sonnei were assessed by ELISA and were detected with goat anti-human immunoglobulin G conjugated to alkaline phosphatase (Sigma Aldrich, A3187).

Validation

NA (primary antibodies were antibodies from the clinical serum samples against S. sonnei LPS).

Clinical data

Policy information about clinical studies

All manuscripts should comply with the ICMJE guidelines for publication of clinical research and a completed CONSORT checklist must be included with all submissions.

Clinical trial registration

NCT03527173

Study protocol

The study protocol is available at www.gsk-studyregister.com (study ID 205626)

Data collection

The study was conducted between August 2018 and November 2019 at the Cincinnati Children's Hospital Medical Center (CCHMC), Ohio, US.

Outcomes

The results of the primary and secondary objectives have previously been reported, including serum anti-S. sonnei LPS IgG levels and SBA titers (reference 33 cited in the manuscript: Frenck, R. W., Jr et al. Efficacy, safety, and immunogenicity of the Shigella sonnei 1790GAHB GMMA candidate vaccine: Results from a phase 2b randomized, placebo-controlled challenge study in adults. EClinicalMedicine 39, 101076 (2021). https://doi.org:10.1016/j.eclinm.2021.101076) This manuscript reports the results of tertiary and exploratory objectives that evaluated S. sonnei specific sIgA in stool, as well as S. sonnei LPS IgA/IgG specific α4β7+/α4β7- ASC plasmablast response pre- and post-vaccination, as indicated in Figure 2 in the manuscript. We also assessed the association with shigellosis for all evaluated immunomarkers, including those previously reported in reference 33, and investigated their role as CoRs. Furthermore, in post-hoc analyses, we evaluated SBA against OAg-positive and OAg-negative Shigella strains after anti-LPS antibody depletion and anti-S. sonnei LPS specific total IgG avidity. In addition, we explored the role of the CoP proposed by Cohen et al. in young Israeli adults in the population of US adults from the CHIM trial. This information is available in the Methods section of the manuscript (Study design and objectives).
